# Knowledge, Applicability, and Barriers of Telemedicine in Egypt: A National Survey

**DOI:** 10.1155/2021/5565652

**Published:** 2021-06-09

**Authors:** Mohamed Alboraie, Mahmoud Abdelrashed Allam, Naglaa Youssef, Mohammad Abdalgaber, Fathiya El-Raey, Nermeen Abdeen, Reem Ezzat Mahdy, Omar Elshaarawy, Ahmed Elgebaly, Tamer Haydara, Sherief Abd-Elsalam, Yusuf Abdullah Nassar, Hosam Shabana, Samy Zaky

**Affiliations:** ^1^Department of Internal Medicine, Al-Azhar University, Cairo, Egypt; ^2^Medical-Surgical Nursing Department, Faculty of Nursing, Cairo University, Cairo, Egypt; ^3^Medical-Surgical Nursing Department, College of Nursing, Princess Nourah Bint Abdulrahman University, Saudi Arabia; ^4^Department of Gastroenterology and Hepatology, Police Authority Hospital, Agoza, Giza, Egypt; ^5^Hepatogastroenterology and Infectious Diseases Department, Al-Azhar University, Damietta, Egypt; ^6^Tropical Medicine, Faculty of Medicine, Alexandria University, Alexandria, Egypt; ^7^Internal Medicine, Assiut University, Assiut, Egypt; ^8^Hepatology and Gastroenterology Department, National Liver Institute, Menoufia University, Menoufia, Egypt; ^9^Faculty of Medicine, Al-Azhar University, Cairo, Egypt; ^10^Internal Medicine, Gastroenterology and Hepatology, Kafrelsheikh University, Kafrelsheikh, Egypt; ^11^Tropical Medicine and Infectious Diseases Department, Tanta University, Tanta, Egypt; ^12^Hepatogastroenterology and Infectious Diseases Department, Al-Azhar University, Cairo, Egypt

## Abstract

**Objectives:**

The study is aimed at evaluating knowledge, attitude, and barriers to telemedicine among the general population in Egypt.

**Methods:**

A questionnaire-based cross-sectional design was carried out among the general Egyptian population. A convenience sampling method was used to approach the eligible participants from University Teaching Hospitals of eight governorates from May to July 2020.

**Results:**

A total of 686 participants filled the questionnaire (49.4% were males, mean age 36.7 ± 11.2 years old). Half of the participants stated that they previously used a telemedicine tool, mainly to follow up laboratory results (67.3%). Video or phone calls (39.3%) and mobile applications (23.7%) were the most commonly recognized telemedicine tools by the participants. The included participants exhibited a high level of knowledge and attitude towards telemedicine. On the other hand, 21.9% stated that telemedicine services could jeopardize patient privacy. 32.8% reported that telemedicine service could lead to disclosing medical information to people who are not authorized to do so. Almost half of the participants agreed to strongly agreed that telemedicine service could increase medical errors. 60.80% of the participants said that they are more likely to prefer telemedicine than traditional ways. However, 13.70% stated that telemedicine is more likely to be challenging to use.

**Conclusion:**

The Egyptian population has high knowledge about the applications of telemedicine. In addition, the vast majority of Egyptians appear to perceive the benefits of telemedicine positively and are willing to use it. However, some barriers that have been found must be taken into consideration to adopt telemedicine successfully, especially for people who are old, are low educated, and live in remote areas. Future studies should address the utility of telemedicine in improving the quality of healthcare and patient's health outcome and quality of life.

## 1. Introduction

The changes in population demography, rising numbers of patients with chronic conditions, and loss of long-term follow-ups encouraged the evolution of novel processes in healthcare [1, 2]. For instance, information and communication technology (ICT) offered promising options to improve healthcare delivery [3]. The use of ICT to facilitate long-distance patient care, preserving patient health records and providing patients with professional aid, is known as telemedicine or e-Health [4, 5]. At the 58^th^ World Health Assembly, the WHO established a telemedicine strategy—regarding it an affordable use of ICT that sustains health and related fields such as healthcare services, screening, education, and research [6].

Healthcare challenges, such as patients' access to cost-effective and high-quality healthcare services, can be overcome by the proper application of telemedicine [7, 8]. A US national survey revealed that around 65% of phone users have downloaded at least one healthcare application [9]. This finding indicates a tendency of the general population towards electronic monitoring of wellbeing and the concept of health at hand [10]. Developed nations utilized this technology by supporting distant monitoring of chronic health conditions and establishing an active online record-keeping system [11].

Meanwhile, the concept of telemedicine remains novel and unclear in the developing world [12, 13]. A study performed in Libya found that only 39% of physicians have a decent understanding of telemedicine, while 12% were unfamiliar with this strategy [14]. The study revealed that physicians' knowledge about telemedicine significantly affected their attitude towards applying this technology [14]. However, literature is rare from Egypt, declaring the need for more research on this topic.

Many reasons could explain why the development and implementation of telemedicine remain challenging in this part of the world. The adoption of any new technology depends on understanding its new concept by users, obtaining its required skills and the suitable working environment. Subsequently, for telemedicine to become adopted into the Egyptian healthcare system, we need to evaluate its knowledge, attitude, and practices among healthcare professionals and the general population.

Accordingly, this study is aimed at evaluating knowledge, attitude, and preference of telemedicine and barriers to its utilization among the general Egyptian population.

### 1.1. Study's Objectives

The study has three objectives:

1. Evaluate telemedicine's knowledge, attitude, and preference among the general Egyptian population
2. Assess the population perception of barriers to applying telemedicine in Egypt
3. Compare knowledge, attitude, preference, and barrier scores according to sociodemographic characteristics

## 2. Materials and Methods

The preparation of the present manuscript runs in compliance with the recommendations of the STROBE statement [15].

### 2.1. Study Design, Settings, and Population

A cross-sectional survey was used to achieve this study's aims. A convenience sampling method was used to approach the eligible participants from University Teaching Hospitals of eight governorates: Cairo, Giza, Tanta, Damietta, Alexandria, Kafrelsheikh, Menoufia, and Assiut. Participants who met these criteria were eligible to participate if they were ≥21 years old and accepted to participate, while healthcare staff (i.e., physicians, nurses, technicians, administrative clerks, and hospital managers) and people who could not fill the questionnaire were excluded from the present survey.

### 2.2. Sample Size Calculation

There are no comparable studies from Egypt that can be used to calculate the required sample size. In a previous report from Karachi, the correct overall rate of the knowledge questionnaire about telemedicine was 80.7% [16]. Thus, the below equation was used to calculate the sample size by assuming a confidence interval level of 95%. A sample size of 270 was determined to be enough.

(1) $$\begin{array}{c} n=\frac{\left[\text{DEFF}*Np\left(1-p\right)\right]}{\left[\left(d^{2}/Z_{1-\alpha /2}^{2}\right)*\left(N-1\right)+p*\left(1-p\right)\right]}, \end{array}$$

where *n* is the sample size, DEFF is the design effect = 1, *N* is population size = 10,000, *p* is the proportion of the outcome = 0.5, *d* is confidence limits = 0.5, and *Z*_1−*a*/2_ = 196.

### 2.3. Measurements

A three-domain Arabic questionnaire was designed after reviewing the literature [16]. The first domain collected the participants' demographic characteristics and previous encounters with a telemedicine tool. The second domain has three subsections: (i) knowledge of telemedicine uses, (ii) attitude towards telemedicine utilization, and (iii) preference for utilizing telemedicine. Participants give their response by using a 5-point Likert scale (1: strongly disagree, 2: disagree, 3: neutral, 4: agree, and 5: strongly agree). To assess the participants' preference of utilizing telemedicine over the traditional way, a visual analog scale from 0 to 10 was used, where 0 meant “I do not prefer it at all” and 10 meant “I prefer it to a great extent.” The third domain evaluated the participants' perception of the barriers to telemedicine utilization.

Four of the research team, experts with a telemedicine and questionnaire structure, checked the questionnaire face and content validity. The questionnaire understandability was examined on a pilot of five participants before data collection.

### 2.4. Data Collection

The questionnaire was distributed from May to July 2020 by face-to-face contact.

### 2.5. Ethical Considerations

The ethical approval of the study (approval number 00208/2020) was obtained from the ethics committee of the National Liver Institute, Menoufia University. Each participant gave his/her written informed consent after the study's objectives were clearly explained. Confidentiality and anonymity of the participants were maintained throughout the study, and their identified data were not collected. They were informed that their participation is voluntary, and they can withdraw from the study at any time.

### 2.6. Statistical Analysis

Data were entered and validated using Microsoft Excel 2019. The *Statistical Package of Social Science* (SPSS) version 22 was used for data analysis. All continuous quantitative data were presented in mean and standard deviation (SD). Categorical data were presented in frequencies and percentages. We used the Student *t*-test or ANOVA test to compare means and the chi-square test to compare frequencies. A *p* value of 0.05 was considered significant.

## 3. Results

### 3.1. Sociodemographic Characteristics

A total of 686 participants filled the questionnaire and the response rate is 66%. Their mean age was 36.7 ± (SD) 11.2 years old, and more than half were female. Most of them (55.5%) were from lower Egypt, and 37.2% were from Cairo. Almost 86% had a university or higher educational level. 35.4% worked at governmental-based facilities, with working hours ranging from six to ten hours in most of them (Table 1).

Almost 23% of the participants had comorbidities, mainly hypertension and diabetes mellitus. Regarding their telemedicine experience, 50.4% of the participants previously used a telemedicine tool, mainly to follow-up their laboratory results (67.3%). Video or phone calls (39.3%) and mobile applications (23.7%) were the most commonly used telemedicine tools by the participants, while mobile applications represented the most preferred tool. The majority of the participants, who had experience with telemedicine, reported that they used it before the COVID-19 pandemic (Table 1).

### 3.2. Knowledge, Attitude, and Preference for Telemedicine

**Figure 1:**
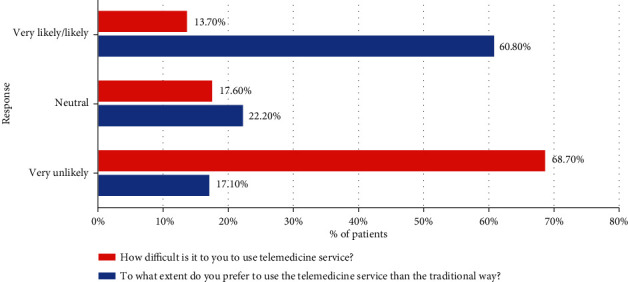
The overall perception towards telemedicine.

Table 2 shows the participants' knowledge and attitude towards telemedicine. Most (73.5%) of the participants agreed to strongly agreed that telemedicine provides faster medical care than traditional approaches. Likewise, 81.9% agreed to strongly agreed that telemedicine is necessary for patient care. The majority of the participants agreed to strongly agreed that telemedicine is essential for delivering medical care to remote and underserved areas of healthcare. The vast majority also agreed that telemedicine saves efforts, money, and transportation cost and reduces hospital waiting lists. Two-thirds of the participants agreed to strongly agreed that telemedicine improves communication between patients and their doctor or nurse. A similar percentage agreed that telemedicine service could help in providing appropriate instructions in emergencies. Overall, 60.80% of the participants said that they are more likely to prefer telemedicine than traditional ways (Figure 1).

### 3.3. Perception of Barriers to Applying Telemedicine in Egypt

On the other hand, only 21.9% stated that telemedicine services could jeopardize patient privacy and 32.8% stated that it could lead to the disclosure of medical information to people who are not authorized to do so. Almost half of the participants agreed to strongly agreed that telemedicine service could increase medical errors. 13.70% stated that telemedicine is likely to be challenging to use (Figure 1).

### 3.4. Knowledge, Attitude, Preference, and Barrier Scores according to Sociodemographic Characteristics

The association analysis demonstrated that unemployed and less-educated participants had less knowledge and less favorable attitude towards telemedicine than other job categories and participants with higher education. Likewise, participants who work for more than 10 hours per day have less knowledge and a less favorable attitude toward telemedical utilization than those who work for less than 10 hours per day (Table 3).

## 4. Discussion

Although telemedicine has emerged as a valuable tool with many applications in diagnosis and treatment, little is known about the level of its utilization and populations' perception in developing countries like Egypt. Thus, we investigated the knowledge and perception of the general Egyptian population towards telemedicine. Overall, Egyptians exhibited a high level of knowledge about the use of telemedicine and its tool. Our survey also demonstrated that many Egyptians use one or more telemedicine tools and have a favorable attitude towards its expansion. The mobile applications represented the most preferred telemedicine tools by Egyptians. On the other hand, some participants exhibited concerns regarding the higher chance of medical errors and violation of patients' privacy when using telemedicine than traditional tools. We found that higher educational levels and current employment were associated with higher knowledge levels and a more favorable attitude towards telemedicine.

Since its initial use in the early 1970s, mainly by military and space institutions, the use of telemedicine has expanded exponentially to cover “the diagnosis of treatment, prevention of disease and injuries, research and evaluation, and education of healthcare providers,” according to the World Health Organization definition [17]. The uses of telemedicine include, but not limited to, scheduling remote visits with treating physicians, remote diagnosis of many diseases according to patients' description of signs and symptoms, remote monitoring of patients with chronic diseases, and remote review of laboratory and imaging findings and teleconsultation [18].

To that end, the use of telemedicine offers many advantages to the healthcare system that range from prompt access to trusted medical information to improving access to better quality healthcare services in limited-resource areas and reduction in healthcare expenses [19]. In developing countries, telemedicine has substantial potentials in improving the quality of care of their population. Besides its advantages mentioned above, telemedicine can reduce the number of needed physicians per population size and relieve some of the needed costs to sustain acceptable healthcare services, especially in countries with limited costs dedicated to the healthcare system [20].

The wide use of telemedicine can overcome improper information systems and enhance treatment compliance among patients with chronic diseases in many developing countries [21]. Besides, telemedicine can be useful during humanitarian disasters by providing a fast and reliable global healthcare network to facilitate the timely delivery of much-needed aids during emergencies [22]. The lack of technological infrastructures was previously considered a primary barrier to telemedicine in developing countries. Recent surveys demonstrated that digital methods of communication, such as mobile phones and the Internet, have become widely available among the general population over the past decade [23]. In Egypt, statistical figures demonstrated that more than 90 million mobile users and nearly 37% of the population were Internet users by 2012 [21]. Many Egyptian initiatives were conducted to implement telemedicine within the healthcare systems, which covered teleconsultation, teleinterpretation of laboratory/imaging tests, and screening for early breast cancer using digital mammography [24].

Nonetheless, the adoption of any new technology depends on understanding its new concept by users, obtaining its required skills and the suitable working environment. Adequate knowledge is crucial to encourage wide use of any new health services by the community. The current body of evidence highlights that good knowledge correlates positively with telemedicine applications [25]. In the present survey, the Egyptian population exhibited adequate knowledge regarding the use and advantages of telemedicine; the level of knowledge was better among employed participants and higher educational levels. To the best of our knowledge, no previous national surveys have been published till carrying out this study about the general population's knowledge and attitude toward telemedicine. However, previous surveys demonstrated that Egyptian physicians had proper knowledge about telemedicine applications [21, 26]. Our results are also in line with other surveys from the Eastern Mediterranean region, such as Saudi Arabia [27, 28].

Population attitude and perception are major driving factors for successfully implanting any healthcare services [29]. Our study findings showed that the Egyptian population has a favorable attitude towards telemedicine expansion. However, legal issues surrounding patient privacy and confidentiality are concerns that may limit the use of telemedicine by a considerable proportion of the population. Moreover, some participants exhibited concerns regarding the higher chance of medical errors and violation of patients' privacy when using telemedicine than traditional tools. Previous studies found that high costs, underdeveloped infrastructure, shortage of technical staff expertise, healthcare provider resistance to change, patient's resistance to change, lack of training on information technology, cultural aspects, legal issues, patient's age, and patient's education level are the main barriers to telemedicine development and adoption globally and particularly in the Middle East region [30, 31]. Therefore, all these factors must be taken into consideration to adopt telemedicine successfully.

To get the most benefit of telemedicine in Egypt, innovative methods must be implemented to facilitate telemedicine service, especially for patients who are old, are without previous telemedicine experience, are low educated, and live in remote areas (out of urban areas). Future studies should address the utility of telemedicine in improving the quality of healthcare and patient's health outcome and quality of life.

The study limitations that must be considered are as follows: developing a causal relationship between the investigated variables was unfeasible because the method of data collection was a snapshot. Also, most of the study's participants were young and half did not use telemedicine before. Many were healthy with no chronic diseases; most of them have a high level of education and living in urban areas. However, our study had an adequate sample that was recruited from eight geographical regions in Egypt, which increases the possibility of generalizing the results to other regions. Therefore, further studies using other research methods and people with different sociodemographic characteristics might yield other results.

## 5. Conclusions

In conclusion, the Egyptian population has high knowledge about the applications of telemedicine. In addition, the vast majority of Egyptians appear to perceive the benefits of telemedicine positively and are willing to use it. The level of knowledge was significantly associated with education and employment; thus, targeted knowledge campaigns should be directed towards the less educated and unemployed population.

## Figures and Tables

**Table 1:** Demographic and behavioral characteristics of the participants (*n* = 686).

Variable	No. (%)
Age, mean ± SD	36.7 ± 11.2
*Sex*	
Male	339 (49.4%)
Female	347 (50.6%)
*Residency*	
Lower Egypt	381 (55.5%)
Upper Egypt	48 (7.0%)
Cairo	255 (37.2%)
Sinai	2 (0.3%)
*Education*	
Postuniversity	192 (28.0%)
University	401 (58.5%)
Preuniversity	81 (11.8%)
Basic	9 (1.3%)
None	3 (0.4%)
*Job*	
Governmental	243 (35.4%)
Nongovernmental	105 (15.3%)
Unemployed	117 (17.1%)
Freelance	97 (14.1%)
Student	106 (15.5%)
Wages	18 (2.6%)
*Work hour*	
0 h	207 (30.2%)
≤5 h	61 (8.9%)
6–10 h	336 (49.0%)
>10 h	82 (12.0%)
*Comorbidities*	
No	530 (77.3%)
Yes	156 (22.7%)
*Previous use of telemedicine services*	
No	340 (49.6%)
Yes	346 (50.4%)
*Reason for using telemedicine*	
Follow-up or showing lab result	237 (67.3%)
Emergency	30 (8.5%)
Quarantine	28 (8.0%)
Multiple	57 (16.2%)
*First telemedicine use*	
Never	280 (40.8%)
After COVID-19	121 (17.6%)
Before COVID-19	285 (41.5%)
*Knowing telemedicine tools*	
Mobile apps	163 (23.7%)
Video or phone calls	271 (39.5%)
Audio chat	74 (10.8%)
Multiple	79 (11.8%)
None	99 (14.4%)
*Preferred telemedicine tools*	
Audio chat	107 (15.6%)
Video or phone calls	142 (20.7%)
Mobile apps	320 (44.6%)
Multiple	15 (2.10%)
None	102 (14.9%)

**Table 2:** Description of knowledge and attitude toward telemedicine among participants (*n* = 686).

Question	1: strongly disagree	2: disagree	3: neutral	4: agree	5: strongly agree
	No. (%)				
(1) Providing a telemedicine service helps faster medical care.	8 (1.2%)	23 (3.4%)	151 (22%)	401 (58.5%)	103 (15%)
(2) Telemedical service may be necessary for patient care.	4 (0.6%)	15 (3.2%)	105 (15.3%)	437 (63.7%)	125 (18.2%)
(3) Providing telemedicine is important for medical care to remote and underserved areas of healthcare.	12 (1.0%)	14 (2%)	68 (9.9%)	394 (57.4%)	198 (28.9%)
(4) Providing a telemedicine service saves effort.	4 (0.6%)	31 (4.5%)	93 (13.6%)	442 (64.4%)	116 (16.9%)
(5) Providing a telemedicine service saves money.	6 (0.9%)	46 (6.7%)	124 (18.15)	403 (58.7%)	109 (15.9%)
(6) Providing a telemedicine service saves transportation cost.	4 (0.6%)	18 (2.6%)	62 (9%)	439 (64%)	163 (23.8%)
(7) Providing a telemedicine service reduces waiting lists in medical centers.	7 (1.0%)	14 (2%)	52 (7.6%)	426 (62.1%)	187 (27.3%)
(8) Providing a telemedicine service can improve communication between patients and their doctor or nurse.	13 (1.9%)	76 (11.1%)	140 (20.4%)	366 (53.4%)	91 (13.3%)
(9) Providing a telemedicine service can help in providing appropriate instructions in emergencies.	11 (1.6%)	26 (3.8%)	72 (10.5%)	385 (56.1%)	192 (28%)
(10) Providing a telemedicine service can jeopardize patient privacy.	41 (6%)	298 (43.4%)	204 (29.7%)	120 (17.5%)	23 (3.4%)
(11) Providing a telemedicine service can lead to disclosing medical information to people who are not authorized to do so.	35 (5.1%)	228 (33.2%)	198 (28.9%)	191 (27.8%)	34 (5%)
(12) Providing a telemedicine service can increase medical errors.	12 (1.7%)	101 (14.7%)	248 (36.2%)	251 (36.6%)	74 (10.8%)
(13) Do you agree to subscribe to an electronic application that tells or warns you if someone had COVID-19?	5 (0.7%)	36 (5.2%)	37 (5.4%)	359 (52.3%)	249 (36.3%)
(14) If you had COVID-19, do you agree to disclose this information to people close to you, through an electronic application for the Ministry of Health?	5 (0.7%)	35 (5.1%)	52 (7.6%)	373 (54.4%)	220 (32.1%)
(15) If you had COVID-19, do you agree to use the electronic application to alert those in contact with you without disclosing your identity?	8 (1.2%)	46 (6.7%)	49 (7.1%)	383 (55.8%)	200 (29.2%)

**Table 3:** Comparisons of knowledge, attitude, preference, and barrier scores according to sociodemographic characteristics (*n* = 686).

Variables	No.	Kn score	*p* value	At score	*p* value	Pref score	*p* value	Barri score	*p* value
		$\overset{¯}{x}\pm \text{SD}$		$\overset{¯}{x}\pm \text{SD}$		$\overset{¯}{x}\pm \text{SD}$		$\overset{¯}{x}\pm \text{SD}$	
Sex			0.890		0.744		0.382		**0.006**
(i) Male	339	74.32 ± 7.78		82.32 ± 13.70		64.9 ± 26.60		26.2 ± 25.94	
(ii) Female	347	74.26 ± 7.18		82.34 ± 12.76		63.8 ± 25.23		31.7 ± 27.15	
Residence			0.301		0.736		0.202		0.379
(i) Lower Egypt	381	74.4 ± 7.44		82.4 ± 13.5		64.0 ± 27.1		30.1 ± 27.5	
(ii) Upper Egypt	48	74.5 ± 7.29		84.0 ± 12.8		65.4 ± 26.8		26.0 ± 25.5	
(iii) Cairo	255	74.1 ± 7.60		81.9 ± 13.0		65.1 ± 23.7		27.7 ± 25.7	
(iv) Sinai	2	67.5 ± 3.54		80.0 ± 0.00		20.0 ± 14.1		50.0 ± 0.00	
Education			**0.015**		**0.006**		**0.011**		**0.006**
(i) Postuniversity	192	72.7 ± 7.70		80.8 ± 14.0		60.1 ± 29.2		31.7 ± 27.2	
(ii) University	401	74.8 ± 7.41		82.9 ± 12.8		65.1 ± 24.3		27.7 ± 26.0	
(iii) Preuniversity	81	75.5 ± 7.11		83.4 ± 13.9		69.6 ± 25.0		28.3 ± 27.7	
(iv) Basic	9	74.4 ± 5.83		81.5 ± 10.4		76.7 ± 21.8		27.8 ± 31.9	
(v) None	3	73.3 ± 3.33		77.8 ± 3.85		63.3 ± 5.77		50.0 ± 36.1	
Job			**0.034**		**0.002**		0.234		0.519
(i) Governmental	243	73.2 ± 7.38		80.2 ± 13.5		63.5 ± 27.8		30.5 ± 28.1	
(ii) Nongovernmental	105	75.0 ± 8.33		84.3 ± 12.5		66.8 ± 25.1		26.6 ± 23.8	
(iii) Unemployed	117	75.2 ± 6.90		81.9 ± 12.1		66.8 ± 22.9		32.3 ± 28.0	
(iv) Freelance	97	74.2 ± 6.66							
(v) Student	106	75.6 ± 7.68		86.3 ± 10.8		59.9 ± 25.2		25.8 ± 24.5	
(vi) Wages	18	71.1 ± 8.28		79.6 ± 9.83		57.8 ± 30.8		30.0 ± 21.1	
Work hours			**0.039**		0.117		**0.010**		0.515
(i) 0 h	207	75.3 ± 7.22		83.7 ± 11.8		64.4 ± 24.3		28.6 ± 26.7	
(ii) ≤5 h	61	73.5 ± 6.10		82.1 ± 14.0		64.1 ± 26.7		33.8 ± 29.4	
(iii) 6–10 h	336	74.4 ± 7.45		82.3 ± 13.6		66.3 ± 26.7		28.4 ± 26.9	
(iv) >10 h	82	72.0 ± 8.63		79.3 ± 14.2		56.3 ± 24.8		28.8 ± 23.5	
Comorbidities			0.585		0.165		0.680		0151
(i) No	530	74.4 ± 7.62		82.6 ± 13.5		64.3 ± 25.2		28.1 ± 26.2	
(ii) Yes	156	73.9 ± 6.98		81.3 ± 12.1		64.6 ± 28.2		32.0 ± 28.2	
Previous use			0.404		0.614		0.256		0.092
(i) No	340	74.5 ± 7.03		82.0 ± 13.4		65.1 ± 26.4		30.9 ± 27.5	
(ii) Yes	346	74.1 ± 7.90		82.6 ± 13.0		63.6 ± 25.4		27.1 ± 25.7	
First use			0.385		0.189		0.389		**0.046**
(i) Never	280	74.4 ± 7.35		82.2 ± 12.7		64.0 ± 26.6		31.8 ± 27.2	
(ii) Before COVID-19	121	75.0 ± 7.39		84.1 ± 13.2		67.4 ± 24.6		25.5 ± 25.3	
(iii) After COVID-19	285	73.9 ± 7.64		81.7 ± 13.6		63.4 ± 25.7		27.8 ± 26.6	

Kn: knowledge; At: attitude; Pref: preference to use telemedicine; Barri: barriers to use telemedicine.

## Data Availability

The datasets used and analyzed during the current study are available from the corresponding author on reasonable request.
